# Visualizing the unusual spectral weight transfer in DyBa_2_Cu_3_O_7–δ_ thin film

**DOI:** 10.1038/s41598-021-04692-9

**Published:** 2022-01-17

**Authors:** Hui Li, Zengyi Du, Ze-Bin Wu, Daniel Putzky, Sang Hyun Joo, Asish K. Kundu, Xiaotao Xu, Xiaoyan Shi, Jinho Lee, Abhay N. Pasupathy, Gennady Logvenov, Bernhard Keimer, Tonica Valla, Ivan Božović, Ilya K. Drozdov, Kazuhiro Fujita

**Affiliations:** 1 Condensed Matter Physics and Materials Science Department, Brookhaven National Laboratory, Upton, NY 11973 USA; 2 Department of Physics and Astronomy, Stony Brook University, Stony Brook, NY 11794 USA; 3 Max Planck Institute for Solid State Research, Heisenbergstrasse 1, 70569 Stuttgart, Germany; 4 Department of Physics and Astronomy, Seoul National University, Seoul, 08826 Republic of Korea; 5 Department of Physics, The University of Texas at Dallas, Richardson, TX 75080 USA; 6 Department of Physics, Columbia University, New York, NY 10027 USA; 7 Department of Chemistry, Yale University, New Haven, CT 06520 USA

**Keywords:** Electronic properties and materials, Superconducting properties and materials

## Abstract

We report a Spectroscopic Imaging Scanning Tunneling Microscopy (SI-STM) study of a DyBa_2_Cu_3_O_7-δ_ (DBCO) thin film (*T*_c_ ~ 79 K) synthesized by the molecular beam epitaxy (MBE). We observed an unusual transfer of spectral weight in the local density of states (LDOS) spectra occurring only within the superconducting gap. By a systematic control of the tip-sample distance and the junction resistance, we demonstrate that the spectral weight transfer can be switched at a nano-meter length scale. These results suggest that an interaction between the STM tip and the sample alters the electronic configurations in the film. This probably originates from a combination of an intrinsic band bending at the interface between the surface and the bulk, and a tip-induced band bending. These results may open a new avenue for band engineering and applications of thin films of high-*T*_c_ cuprates.

## Introduction

YBa_2_Cu_3_O_7-δ_ (YBCO) is one of the most studied high *T*_c_ cuprate superconductors, because large single crystals are relatively easily obtained and doping levels are readily varied by removing oxygen atoms from CuO chains. The electronic structure of the YBCO has been studied extensively, and its temperature-hole density phase diagram has been well established, exhibiting a variety of states such as antiferromagnetism, charge-density waves (CDW)^1^, intra-unit-cell nematicity^2^ in the enigmatic pseudogap state, as well as d-wave superconductivity. A recent development of the resonant x-ray scattering technique further revealed a giant phonon anomaly at the similar wavevector to that of the CDW^3^, appearing near the pseudogap opening temperature T*. However, the CDW order is detected^4^ only well below T*, while the nematicity onsets^5^ at T*. Thus, one possible scenario is that the pseudogap state originates from intra-unit cell nematicity. However, a caveat is that in general, ***Q*** = 0 order (nematicity) should not open a gap at the Fermi surface. On the other hand, no gap opening has been detected at “*T*_cdw_”, although CDW is indeed expected to open a gap at the Fermi surface. A recently proposed concept of “vestigial nematicity” may resolve these apparent contradictions^6^. An alternative explanation postulates that the pseudogap originates from a pair-density wave state, which could also give rise to the vestigial nematicity^7,8^. Understanding these issues has been a focus of intense recent studies of cuprates.

YBCO is a convenient material choice to study the questions posed above. Indeed, it would be highly beneficial to study YBCO^9,10^ or its variant (Re-123 cuprates)^11,12^ by surface sensitive spectroscopic tools, such as angle resolved photoemission spectroscopy (ARPES) and SI-STM, to find out commonalities among different cuprate families, and/or new functionalities in the electronic structure, if any. However, it has been challenging to study electronic structure of YBCO by these techniques: an exposed surface after the cleaving is not reproducible, which is either BaO or CuO chain layers. In addition, the metallic CuO chain scrambles the electronic structure of the CuO_2_ plane. For these reasons, studies by (ARPES) and SI-STM on this cuprate family^13,14^ are far less abundant than those for the bismuth-based cuprates.

In this study, we use a multi-modal approach of combining the MBE and SI-STM techniques, in which a DyBa_2_Cu_3_O_7-δ_ (DBCO) thin film is synthesized by the atomic layer-by-layer MBE technique and transferred *in-situ* to the SI-STM without breaking vacuum. In this approach, the DBCO sample surface is not exposed to atmosphere and contaminated, and immediate morphological and electronic information is obtained by the SI-STM measurements. This type of approach has already demonstrated a great advantage in in-situ studies of both the surface and the electronic structures^15–19^. In fact, our previous study of DBCO thin film, in which the same approach was used, revealed highly homogeneous superconducting gap at low energies, while the so-called pseudogap observed at relatively higher energies are strongly heterogeneous^20^. A systematic study and more detailed analysis on this film further revealed intriguing phenomena related to a local band bending as we shall demonstrate below.

## Results

### Unusual LDOS evolution on DBCO film

**Figure 1:**
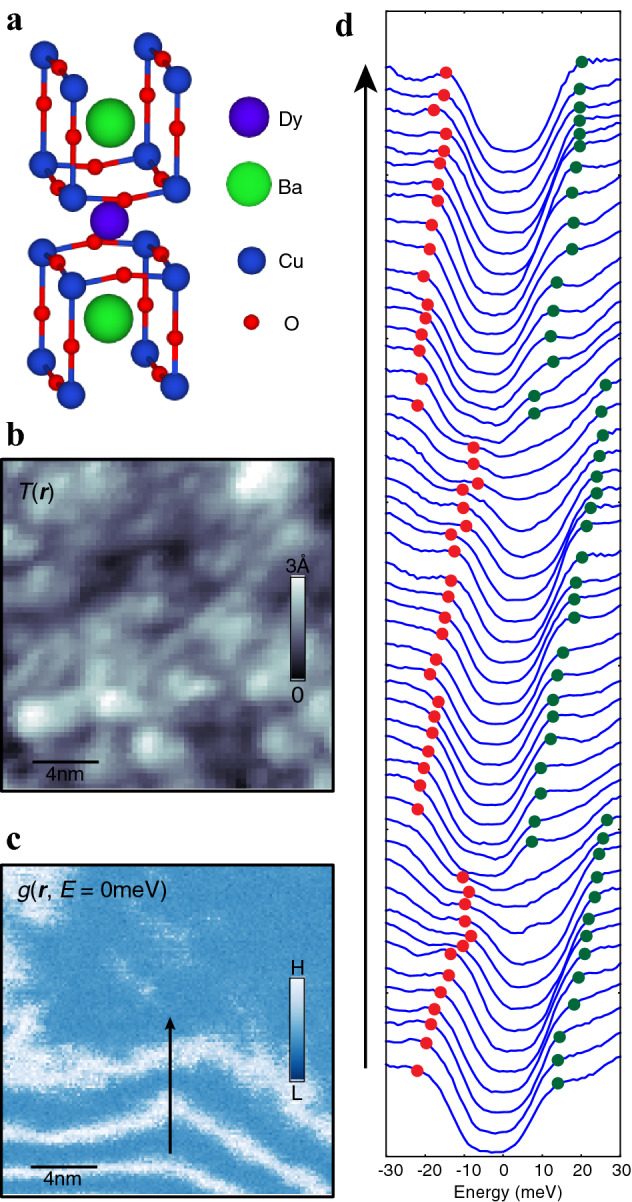
DyBa_2_Cu_3_O_7-δ_ topography and LDOS spectra. (**a**) Crystal structure of DyBa_2_Cu_3_O_7-δ_. (**b**) Typical STM topography of a 20 nm $\times$ 20 nm FOV, measured at *V*_bias_ = 150 mV and *I* = 50pA. (**c**) Differential conductance map, *g*(***r***, *E* = 0 meV) in a 20 nm $\times$ 20 nm FOV measured at *V*_bias_ = 150 mV and *I* = 150pA. (**d**) A line cut through the black solid arrow in **c**. The spectral shift in energy is obvious. Red and green solid dots indicate the negative and positive energy peak/kink positions of the superconducting gap energies.

The crystal structure of DBCO, shown in Fig. 1a, is the same as in YBCO. A mutual inductance measurement showed that the superconducting transition temperature (*T*_c_) of the present film is 79 K, implying that the film is slightly underdoped. The MBE-synthesized DBCO film was transferred into the STM *in-situ* under an ultra-high vacuum, and SI-STM measurements were carried out at *T* = 9 K using a tungsten tip. The differential conductance spectrum *g*(***r***, *E*) $\equiv$ d*I*/d*V*(***r***, *E*), which is proportional to the local density of states (LDOS), is measured in the field of view of the interest, yielding a spatial LDOS map as a function of the bias voltage, simultaneously with the topography.

Figure 1b is a typical topography of the DBCO thin film, in the field of view of 20 nm $\times$ 20 nm. Individual atoms are not clearly resolved but local atomic displacements as a result of the loss of the oxygens are visible, indicating a surface reconstruction. Note that variations of the height are within 3 Å so the surface is in fact rather flat. Interestingly, as shown in Fig. 1c, the *g*(***r***, *E*) map taken at another 20 nm x 20 nm FOV shows spatial variations that are not related to the features in the simultaneously measured topography (Supplementary Fig. 1 and Ref. 22). Instead, the *g*(***r***, *E*) map shows unusual “saw-tooth” shaped line-segment structure that changes with the bias voltage. Figure 1d shows the spatial variations in the *g*(***r***, *E*) spectra extracted along the line indicated by the black arrow in Fig. 1c. All spectra show gap-like features, and we assign the peak/kink position as the superconducting gap $\Delta$. The spectral weight transfer shows striking modulation as we move along the black line. The red (green) solid circles represent the peak/kink positions at negative (positive) bias voltages, ${\Delta }_{-} ( {\Delta }_{+})$. Upon traveling across the black line in Fig. 1c, both ${\Delta }_{-} \mathrm{and} {\Delta }_{+}$ vary, shifting in energy together with the minimum in *g*(***r***, *E*). It is also evident in Fig. 1d that a second and third sets of such spectral transfer occur when the next white line segment is crossed. These phenomena can be viewed as an energy offset in the LDOS spectra, but it should be noted that these happen only below the superconducting gap energy scales $\left| {\Delta_{ - } } \right|\;{\text{and}}\;\left| {\Delta_{ + } } \right|$, while an equivalent spectral weight shift at high energies is not present. Rather, it shows an electronic heterogeneity related to the pseudogap^20^ (Supplementary Fig. 2).

### Gap maps and the spectral weight shift

**Figure 2:**
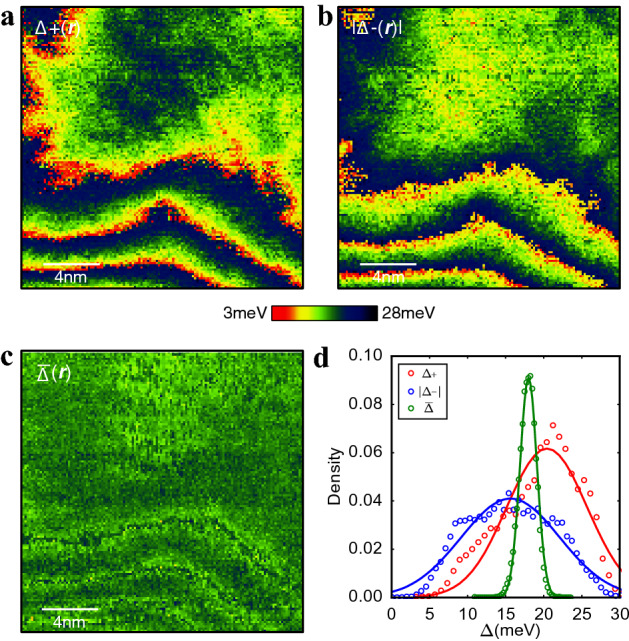
Gap maps and gap distributions. (**a** and **b**) Positive and negative energy gap maps. There is a clear anti-correlation between them. (**c**) Average gap map defined as $\left( {\Delta_{ + } + \left| {\Delta_{ - } } \right|} \right)/2$ , representing extremely uniform spatial variations. (**d**) Distributions of three gaps from a, b, and c. Solid curves are obtained by gaussian fittings to each distribution. The mean values for $\Delta_{ + }$, $\left| {\Delta_{ - } } \right|$, and $\overline{\Delta }$ are 20.4 meV, 15.6 meV and 18.1 meV, respectively.

Now, we examine the gap values for both bias polarities, ∆_+_ and ∆_−_, of this DBCO film. For each bias polarity, the local gap value is defined as an energy where the LDOS spectrum is peaked or kinked. The peaks or kinks are typically found below |*E*|< 30 meV. We extracted the ∆_+_ and ∆_−_ values from spectra in the entire field of view, constructing the ∆_+_(***r***) and ∆_−_(***r***)“gap maps”, as shown in Fig. 2a and b, respectively. Following the spectral evolution in Fig. 1d, the two gap maps show spatial variations quite similar to those of *g*(***r***, *E*) as in Fig. 1c. However, ∆_+_(***r***) and ∆_−_(***r***) are strongly anti-correlated; when ∆_+_(***r***) is higher, ∆_−_(***r***) is lower, and vice versa. This observation may appear inconsistent with observation that the gap size is spatially uniform, as reported in a previous study^20^. However, if we define the gap 2 ${\Delta }$ as the separation of the “coherence peaks”, then the map of ${\Delta }\left( {\varvec{r}} \right) = \left( {\Delta_{ + } \left( {\varvec{r}} \right) + \Delta_{ - } \left( {\varvec{r}} \right)} \right)/2$ is indeed quite uniform in space, as seen in Fig. 2c. Figure 2d shows the distributions of ∆_+_(***r***), ∆_−_(***r***), and ${\Delta }\left( {\varvec{r}} \right)$ (open circles), together with Gaussian fitting (solid lines). One can see from these histograms that the ${\Delta }\left( {\varvec{r}} \right)$ distribution is much sharper than those of ∆_+_(***r***) and ∆_−_(***r***). The spatial averages of ∆_+_(***r***), ∆_−_(***r***) and ${\Delta }\left( {\varvec{r}} \right)$ are 20.4 meV, 15.6 meV, and 18.1 meV, respectively. These results, together with the anti-correlation of ∆_+_(***r***) and ∆_−_(***r***), indicate that the intrinsic superconducting gap is in fact quite homogeneous in DBCO as reported before^20^. More importantly, the strong anti-correlation of ∆_+_(***r***) and ∆_−_(***r***) and the spatially homogeneous ${\Delta }\left( {\varvec{r}} \right)$ indicate that below |*E*|< 30 meV the LDOS spectra indeed shift back and forth along the energy axis.

The shift of the LDOS and gap energies ∆_+_(***r***) and ∆_−_(***r***) is similar to what is often seen in metal–insulator-semiconductor junctions as a result of local band bending that occurs at an interface of the surface and the bulk of sample^21–24^. Usually, when such a junction is formed, a charge transfer occurs between the surface and the bulk of semiconductor, in order to satisfy the charge neutrality requirement. Then, redistributed local charges at the surface induce band bending over the Debye wavelength, since the carrier concentration is very low, and the screening effect is weak. We have already shown that in the DBCO films the surface layer is much more underdoped than the bulk^20^. We also know that the surface of DBCO is indeed reconstructed, as shown in Fig. 1b. These findings point to redistribution of electrons and/or holes between the surface and bulk of DBCO, and hence one may expect the effect of band bending on the surface of DBCO. However, band bending alters the *g*(***r***, *E*) spectra in such a way that the observed gap is always enhanced on both positive and negative bias voltages^25–27^, while we observe a shift of the *g*(***r***, *E*) spectra in energy with anti-correlation between ∆_+_(***r***) and ∆_−_(***r***). Hence, our observations cannot be simply explained by the effect of the band bending that may be present on the surface of the DBCO film.

It is also natural to consider that, in addition to the “intrinsic” band bending occurring between the surface and bulk of DBCO, there might be a tip-induced band bending (TIBB) effect such that the STM tip and surface separated by vacuum form a junction, and an electric field (or bias voltage) applied across the junction may induce additional band bending in the sample, since the surface of DBCO is not very metallic. Actually, the effect of TIBB has been observed in *g*(***r***, *E*) in previous SI-STM measurements not only in semiconductors^28,29^, but also in strongly correlated materials such as cuprates^30^ and iridates^31^. Hence, it is conceivable that the unusual spectral shift can be due to a combination of the intrinsic band bending and the TIBB. The carriers at the DBCO surface can be redistributed by the formation of a tunnel junction. In this case, a string-like feature seen in Fig. 1c is likely to be caused by the band bending in the vicinity of some localized charges.

### Junction resistance dependent electronic structure

**Figure 3:**
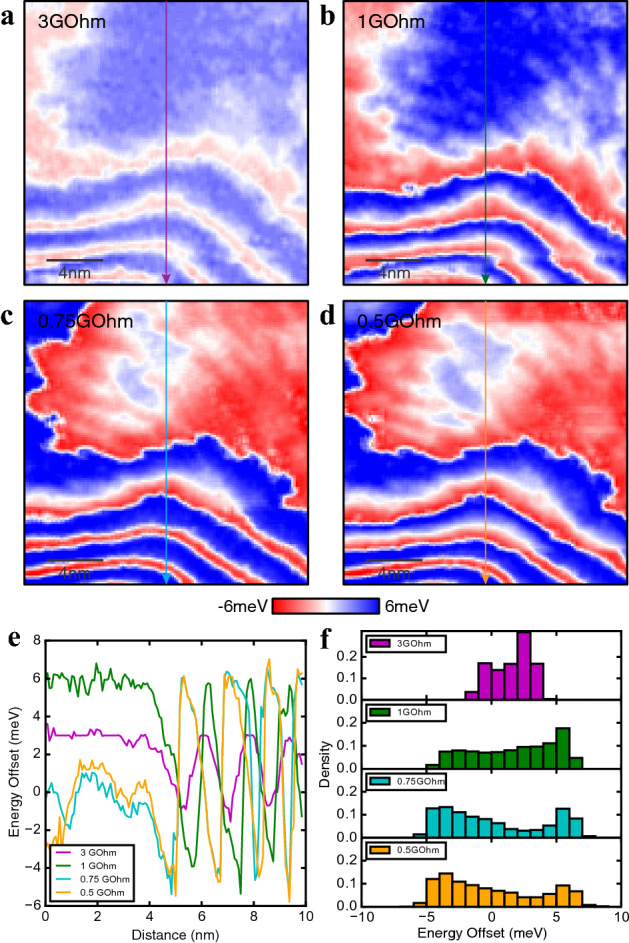
Energy offsets at different junction resistances. (**a–d**) Energy offset maps at 3GOhm, 1GOhm, 0.75GOhm and 0.5GOhm junction resistance in the same FOV. Red (blue) represents spectral shift toward negative (positive) energies. (**e**) Line cuts through the same vertical lines from a, b, c, and d. Magnitudes and phase differences are strongly junction resistance dependent. (**f**) Energy offset distributions of all four junction resistances maps. The energy offsets tend to be larger when junction resistance is smaller, which means tip is closer to the sample.

In order to test this hypothesis, we have measured the *g*(***r***, *E*) maps at different junction resistances, i.e., different tip-sample distances, keeping the field of view unchanged. Note that in the present sample, no strong electronic modulations such as the CDW were clearly observed, which often causes the setup effect in the measurements (spatially dependent tip-sample distances). Here, the electronic structures measured at different setup conditions are virtually identical, indicating that the tip-sample distances are the same everywhere in the FOV (Supplementary Fig. 3). This enables us to perform systematic measurements at different junction resistances without systematic errors. The repeatability of the data taken from the same scanned region is confirmed from the simultaneous topographies, indicating that the STM tip condition is the same for these measurements (Supplementary Fig. 1). Here, we examine the energy offset that is defined as an energy difference between the zero bias and the energy where the minimum in *g*(***r***, *E*) is identified, constructing energy offset maps *O*(***r***) for all the junction resistances. The minimum of *g*(***r***, *E*) is determined by a polynomial fit to the spectrum (Supplementary Fig. 4). Figure 3a-d show *O*(***r***) maps at 3 GOhm, 1 GOhm, 0.75 GOhm and 0.5 GOhm, respectively. *O*(***r***) exhibits spatial variations as expected from the Fig. 1d, and its magnitude is about ± 3 meV for 3 GOhm. The variation of the *O*(***r***) tends to be bigger as the width of the *O*(***r***) distribution increases with decreasing the junction resistance (Fig. 3f). The zig-zag “line segment” features are again present in all maps, and they evolve as a function of junction resistance in a systematic way. One can notice that the spatial variation in *O*(***r***) dramatically changes between 1 GOhm and 0.75 GOhm junction resistances, as the blue regions in Fig. 3b are now flipped to red in Fig. 3c, and vice versa, indicating that the energy offset switched the sign. This is also evident in Fig. 3e, where the four line-cuts along the same vertical lines as in Fig. 3a–d are plotted together. These observations, together with the ∆(***r***) distributions shown in Fig. 2, demonstrate that the interaction between the tip and sample is indeed present. More importantly, the electronic structure of DBCO thin film can be modified by controlling the distance between the STM tip and the surface. One can suppose that some localized charges may exist, which are strongly pinned by local disorder at the film surface. This could cause band-bending near the potential center. In addition, the strength of the charge potential may be affected by the unscreened electrical field between the tip and the surface. Obviously, if the tip-sample interaction affects the magnitude of band bending, the energy offset could have a larger variation as the STM tip gets closer to the surface and the junction resistance gets lower.

**Figure 4:**
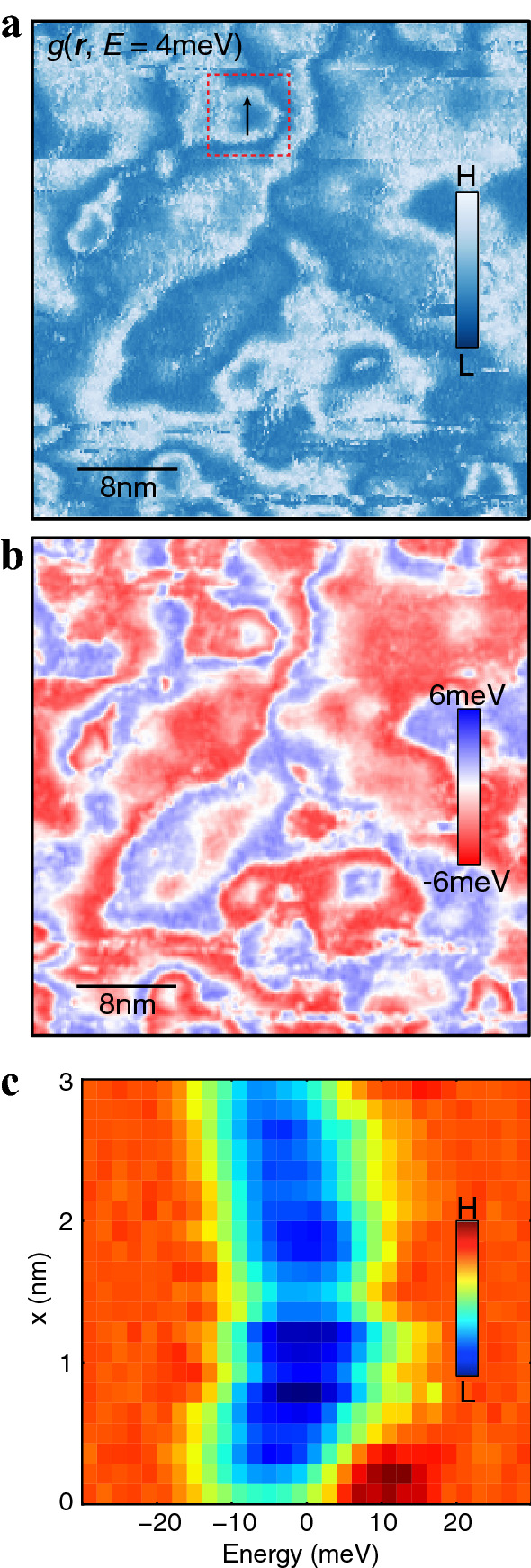
Energy offsets in a larger FOV. (**a**) *g*(***r***, *E* = 4 meV) in a 40 nm $\times$ 40 nm FOV, taken at *V*_bias_ = 150 mV and *I* = 150pA. (**b**) Energy offset map in the same FOV as **a**. Isolated “ring” features are seen in both differential conductance map and energy offset map. (**c**) Evolution of the spectra through the black vertical line in **a** plotted in a two-dimensional intensity plot. Colors represent the LDOS magnitudes.

To investigate a large-scale spatial variation of the TIBB, we have measured the g(***r***, *E*) maps within a larger field of view, 40 nm × 40 nm. Figure 4a shows the *g*(***r***, *E* = 4 meV) map within the gap, in which several rings and even larger-scale features are observed. Such a complicated electronic structure implies a presence of multiple charge potential centers within the field of view, with the net potential resulting from a superposition of the potentials arising from local charge centers. This may indeed result in different sizes and shapes of real-space electronic features. If this is the case, then one would expect a canonical ring-like structure in *g*(***r***,*E*) in the vicinity of a single isolated charge potential center. In fact, we can find such structures; one is highlighted in the box in Fig. 4a. In this area, the ring disperses with increasing bias voltages (see Supplementary Fig. 5), indicating that the spectral shift occurs in a symmetrical fashion with respect to the ring center. In Fig. 4b this is also seen in the *O*(***r***) map, and in Fig. 4c in the line profile along the black solid line in Fig. 4a. As observed in Figs. 2 and  3, the energy-offset map has a strong spatial correspondence with the *g*(***r***, *E* = 4 meV) map, and the line profile in Fig. 4c represents a symmetric parabolic energy dependence as a function of the position. This large FOV map, together with additional measurements (Supplementary Fig. 6 and 7) performed on a different DBCO film that is synthesized by the same conditions, confirms that the spectral weight shift is highly reproducible.

## Summary

By studying a slightly underdoped DBCO thin film (*T*_c_ = 79 K) synthesized by MBE, we have confirmed that DBCO shows the same phenomenology in the local density of states as that in Bi-based cuprates, in which a low-energy superconducting gap and a high-energy pseudogap are observed. New here, in DBCO we have observed unusual spectral weight transfers in the LDOS spectra. The energy offsets of the LDOS spectra occur between positive and negative bias voltages and move back and forth within the superconducting gap energy scale when crossing “ring-like” structures, and the spectral weight transfer can be changed by controlling the distance between the STM tip and the film. We attribute these observations to a combination of the charge re-distribution occurring at the surface of the film and a possible tip-induced band bending due to the interaction between the tip and the sample, modifying the local electronic structure. While similar behavior has been reported in semiconductors, we have observed for the first time that spectral weight transfer in the electronic structure occurs at the superconducting gap energy scales. These features may point toward novel applications of the cuprate films in high-*T*_*c*_ superconducting electronic devices.

## Methods

### MBE synthesis of DyBa_2_Cu_3_O_7-δ_ thin films and ***T***_c_ measurements

The DBCO thin film was synthesized within the MBE module of the OASIS (**O**MBE-**A**RPES-**SIS**TM) system^32^ in Brookhaven National Laboratory. The Nb-doped SrTiO_3_ substrate was polished perpendicular to the crystallographic [001] direction. With ultrahigh vacuum of 8 × 10^−10^ Torr in MBE chamber before the synthesis, ozone is introduced during the growth and the background pressure is set at 3 × 10^−5^ Torr. After calibration of the deposition rate of each elemental source (Dy, Ba, and Cu) by a quartz crystal microbalance, a 20-unit-cell-thick DBCO film was epitaxially synthesized layer-by-layer by source shuttering, while the substrate was heated up by an infra-red source from the back side. *T*_c_ of the sample was determined by *ex-situ* mutual inductance technique after the *in-situ* SI-STM study.

### SI-STM measurements and data analysis

After the synthesis, DBCO film was transferred to the STM module without breaking the UHV environment, and inserted into the STM head after cooling it down. All the measurements were performed at 9 K. For the measurements of the dependence on junction resistance, topographies and *g*(***r, E***) maps were obtained within the 20 nm $\times$ 20 nm field of view, at the setup conditions (*V*_*s*_ = 150 mV, *I*_*s*_ = 50 pA), (150 mV, 150 pA), (150 mV, 200 pA) and (150 mV, 300 pA) for 3 GOhm, 1 GOhm, 0.75 Ohm and 0.5 GOhm, respectively. The gap values were extracted by a Gaussian fitting to each spectrum, after the background subtraction, and the distributions of different gap maps were normalized and then fitted by a Gaussian function.

## Supplementary Information

Supplementary Figures.

## Data Availability

The data sets that support the findings of this study are available from the corresponding author upon a reasonable request.
