# Supplementary material for: Visualizing the unusual spectral weight transfer in DyBa2Cu3O7–δ thin film
Source: Sci Rep. 2022 Jan 17;12:830. doi: 10.1038/s41598-021-04692-9 (PMC8763906; doi:10.1038/s41598-021-04692-9)
Supplement: Supplementary file 1 — Supplementary Figures. [file 41598_2021_4692_MOESM1_ESM.docx]

Supplementary Information for:

**Visualizing the unusual spectral weight transfer in DyBa_2_Cu_3_O_7-δ_ thin film**

Hui Li^1,2^, Zengyi Du^1^, Ze-Bin Wu^1^, Daniel Putzky^3^, Sang Hyun Joo^4^, Asish K. Kundu^1^, Xiaotao Xu^5,1^, Xiaoyan Shi^5^, Jinho Lee^4^, Abhay N. Pasupathy^1,6^, Gennady Logvenov^3^, Bernhard Keimer^3^, Tonica Valla^1^, Ivan Božović^1,7^, Ilya K. Drozdov^1^, Kazuhiro Fujita^1*^

*^1^Condensed Matter Physics and Materials Science Division, Brookhaven National Laboratory, Upton, NY 11973, USA*

*^2^Department of Physics and Astronomy, Stony Brook University, Stony Brook, NY 11794, USA*

*^3^Max Planck Institute for Solid State Research, Heisenbergstrasse 1, Stuttgart 70569, Germany*

*^4^Department of Physics and Astronomy, Seoul National University, Seoul 08826, Republic of Korea*

*^5^Department of Physics, The University of Texas at Dallas, Richardson, Texas 75080, USA.*

*^6^Department of Physics, Columbia University, NY, USA*

*^7^Department of Chemistry, Yale University, New Haven, CT 06520, USA*

**Supplementary Figure 1.** **STM topographies at different junction resistances.** **a** – **d**, Topographies simultaneously taken with *g*(***r***,*E*) maps at 3 GOhm, 1 GOhm, 0.75 GOhm and 0.5 GOhm junction resistances, respectively. The red crosses mark positions of the highest intensity location on a specific topographic feature, with the coordinate in pixel (total FOV size is 128 x 128 pixels). Four maps are taken on a same FOV within 3 pixels error.

**Supplementary Figure 2. Comparison of LDOS maps at low energy and high energy. a** and **b**, differential conductance map at *E* = 5meV and *E* = 90meV, respectively. A setup condition for these maps is *V*_bias_ = 150mV / *I* = 150pA. The spectral weight shifts represented by the white strings at 5meV does not exist at 90meV, which is the pseudogap energy scale.

**Supplementary Figure 3. Setup condition dependence.** **a** and **b**, differential conductance maps at *E* = 10meV measured at tunneling junction setups, *V*_bias_ = 150mV / *I* = 150pA and *V*_bias_ = -150mV / *I* = 150pA, respectively. **c** and **d**, differential conductance maps at *E* = -10meV measured at tunneling junction setups *V*_bias_ = 150mV / *I* = 150pA and *V*_bias_ = -150mV / *I* = 150pA, respectively. a and b (c and d) are virtually identical so that no systematic error arising from an electronic heterogeneity is present.

**Supplementary Figure 4. Procedure to find an energy shift.** The black curve shows a typical d*I*/d*V* spectrum measured on the DBCO sample. The red curve is a polynomial fitting in the energy range between -18meV to 12meV. The energy at the minimum in the polynomial fit is extracted and this procedure is repeated over the entire FOV to construct an energy shift map *O*(***r***).

**Supplementary Figure 5.** *g*(***r***, *E*)  **maps in a 40nm** $\boldsymbol{\times}$**40nm FOV.** The *g*(***r***, *E*) maps at six representative energies from -12meV to 8meV. As highlighted in the red dashed circle, the canonical “ring” structure shrinks with increasing energies. Similarly for other regions.

**Supplementary Figure 6.** **Spectral weight shift observed in another** **sample within a 20nm FOV.** **a**, topography taken at *V*_bias_ = 100mV / *I* = 50pA. **b**, differential conductance map at *E* = 0meV. **c**, energy offset map as described in main text. Majorities of the measured energy offsets are within $\pm4\mathrm{meV}$. **d**, the linecut through the black line in **b**. The dashed black line at *E* = 0meV guides the observation of energy offset.

**Supplementary Figure 7. Spectral weight shift observed in the same sample as in the main figures, but another region within a 50nm FOV.** **a**, topography taken at *V*_bias_ = 100mV / *I* = 100pA. **b**, differential conductance map at *E* = 0meV. **c**, energy offset map as described in main text. Majorities of the measured energy offsets are within $\pm2\mathrm{meV}$. **d**, the linecut through the black line in **b**. The dashed black line at *E* = 0meV guides the observation of energy offset.
